# Correction: *cfTrack:* A Method of Exome-Wide Mutation Analysis of Cell-free DNA to Simultaneously Monitor the Full Spectrum of Cancer Treatment Outcomes Including MRD, Recurrence, and Evolution

**DOI:** 10.1158/1078-0432.CCR-26-0515

**Published:** 2026-03-16

**Authors:** Shuo Li, Weihua Zeng, Xiaohui Ni, Yonggang Zhou, Mary L. Stackpole, Zorawar S. Noor, Zuyang Yuan, Adam Neal, Sanaz Memarzadeh, Edward B. Garon, Steven M. Dubinett, Wenyuan Li, Xianghong Jasmine Zhou

In the original version of this article (1), the disclosures for M.L. Stackpole were not included. In addition, coauthors S. Li, W. Zeng, X. Ni, Y. Zhou, S.M. Dubinett, W. Li, and X.J. Zhou provided more details about the relationships listed in their published disclosures. The error has been corrected and the updates are included in the latest online and PDF versions of the article. The authors regret the error.
